# Meeting open science needs at *PLOS Mental Health*

**DOI:** 10.1371/journal.pmen.0000143

**Published:** 2024-09-18

**Authors:** Karli Montague-Cardoso, Marcel LaFlamme

**Affiliations:** 1 PLOS: Public Library of Science, Cambridge, United Kingdom; 2 PLOS: Public Library of Science, San Francisco, California, United States of America; PLOS: Public Library of Science, UNITED KINGDOM OF GREAT BRITAIN AND NORTHERN IRELAND

Open Science practices support an inclusive research ecosystem in which verifiable knowledge can be produced and shared across distances and disciplines. Yet given the differences in how research communities develop, understand, and enact knowledge [1], the most effective and meaningful efforts to advance Open Science consider community-specific needs and assets [2]. We need to identify any problems associated with sharing across the research cycle that researchers agree are worth solving. At the same time, we need to learn from community-specific practices that have already proven fruitful.

This consultative approach to Open Science can be seen as a defining feature of *PLOS Mental Health*. Indeed, prior to the journal’s launch in November 2023, PLOS conducted a global survey of mental health researchers to begin to understand the Open Science needs of different communities in the field. While the volume of completed responses was modest, this initial assessment has provided some preliminary indications of needs and considerations that will inform how we do things as a journal moving forward. In keeping with the ethos and mission of *PLOS Mental Health*, we will continue to monitor needs through a variety of channels and will adapt when and where necessary, rather than imposing inflexible, one-size-fits-all solutions. We also recognise that it is not possible to meet the needs or priorities of all communities equally at any one time, and so we intend to align our Open Science efforts with our journal priorities—specifically, early career researchers and those from underrepresented demographics.

The needs assessment survey gathered data from just over 200 respondents, across career stages and regions, working in the field of mental health research [3]. Respondents reported being only moderately satisfied with their ability to engage in Open Science practices, given the resources at their disposal. For instance, 80% of our respondents have not shared sensitive research data. In some cases this is due to sensitive data not being part of their research. However, among researchers who do use sensitive data, the decision not to share is often due to ethical and consent restrictions. Other reasons that were given pointed to an overall lack of familiarity with sensitive data sharing. Publishers like PLOS can, to some extent, help to address these barriers—whether by actively facilitating data sharing or by providing guidance to authors. For instance, consent forms and processes can be designed to include permission to share de-identified data [4], while controlled-access repositories can screen data requestors for the nature of their intended reuse [5]. We see a role for *PLOS Mental Health* in connecting researchers with resources like these, while listening closely to those who may still perceive that the risks of sharing outweigh the benefits.

Our survey also indicates that usage of preprints by mental health researchers is relatively low, a result corroborated by the large-scale Open Science Indicators dataset that PLOS maintains [6]. Some respondents noted that posting preprints can be time consuming, a deterrent especially when the research being shared is not viewed as particularly urgent. However, facilitated posting to trusted preprint servers is one way that PLOS can help to address this barrier. Informed by the list of preprint servers to which our survey respondents did report posting, we plan to explore opportunities to bring this convenient service to *PLOS Mental Health*. We also want to ensure that communication surrounding existing Open Science options is consistent, clear and accessible.

As mentioned above, *PLOS Mental Health* aims to prioritise the Open Science needs of authors and readers based in regions that have historically been excluded from the global science system. One interesting finding from the survey is that only 7% of respondents based in the US and Canada view preregistration of studies as extremely important, whereas among our respondents based in Africa, the proportion that hold this view is one-third—despite low to moderate rates of self-reported study registration behaviour by the relatively small sample of respondents from Africa. The apparent importance placed on this Open Science practice by African researchers and practitioners points toward the possible value of Registered Reports as a possible article type on *PLOS Mental Health* that could be considered in the future as the journal matures. In addition, given that one purpose of Registered Reports is to eliminate bias against negative results, offering this article type can contribute to a more inclusive research landscape.

Other trends became visible when segmenting the survey data by career stage. For instance, our results point to protocol sharing as an Open Science practice that holds more importance for early career researchers than for their more senior counterparts (see also [7]). One could speculate that this is partly due to early career researchers being more closely involved with developing the methodological details that protocols document without, at present, receiving credit for doing so. To this end, PLOS has developed peer-reviewed protocol article types that are currently offered on *PLOS ONE* and may be suitable for extension to journals like *PLOS Mental Health* in the future.

In this editorial, we have set out to transparently communicate our initial efforts to understand the current Open Science needs of mental health communities and researchers. Our analysis of the needs assessment survey will pave the way for future efforts to square our Open Science posture as a journal with our overall mission and commitment to serving our communities. Here and elsewhere, it is crucial to understand the areas of opportunity where we can improve the experience—and relevance—of engaging in Open Science practices for our authors.
