# Correction: Meeting open science needs at *PLOS Mental Health*

**DOI:** 10.1371/journal.pmen.0000205

**Published:** 2024-12-05

**Authors:** The PLOS Mental Health Staff

## Notice of republication

This article was republished on November 26, 2024, to correct the capitalization of the journal name in the title. The publisher apologizes for the errors. Please download this article again to view the correct version. The originally published, uncorrected article and the republished, corrected articles are provided here for reference.

## Supporting information

S1 File Originally published, uncorrected article. (PDF)

S2 File Republished, corrected article. (PDF)
